# Effect of HIV status and retinol on immunogenicity to oral cholera vaccine in adult population living in an endemic area of Lukanga Swamps, Zambia

**DOI:** 10.1371/journal.pone.0260552

**Published:** 2021-12-02

**Authors:** Charlie Chaluma Luchen, John Mwaba, Harriet Ng’ombe, Peter Ibukun Oluwa Alabi, Michelo Simuyandi, Obvious N. Chilyabanyama, Luiza Miyanda Hatyoka, Cynthia Mubanga, Samuel Bosomprah, Roma Chilengi, Cleopatra Caroline Chisenga

**Affiliations:** 1 Enteric Diseases and Vaccine Research Unit, Centre for Infectious Disease Research in Zambia (CIDRZ), Lusaka, Zambia; 2 Amsterdam UMC, University of Amsterdam, Institute for Infection and Immunity, Amsterdam, the Netherlands; 3 University of Zambia, School of Health Sciences, Lusaka, Zambia; 4 Department of Biostatistics, School of Public Health, University of Ghana, Accra, Ghana; Emory University School of Medicine, UNITED STATES

## Abstract

**Background:**

We set out to assess the impact of human immunodeficiency virus (HIV) and micronutrient deficiency as indicated by serum retinol levels on the immune responses to Oral Cholera Vaccine (Shanchol™) in a cohort of participants in Lukanga Swamps, Zambia. Cholera remains endemic in Zambia with vaccines being the only effective preventive measures. However, the effect of these vaccines on populations living with HIV has not been widely documented.

**Methods:**

HIV testing and confirmation was done using the Alere Determine™ HIV-1/2 and Uni-Gold™ kits while vibriocidal antibody assay was applied for vaccine immunogenicity. Serum retinol analysis was assessed by Shimadzu Prominence HCT-2010 High Performance Liquid Chromatography (HPLC). The primary outcome was log transformed geometric mean titre.

**Results:**

From 47 participants screened for HIV, 51% (24) tested positive. There was a statistically significant reduction in Ogawa geometric mean ratio (GMR) by 67% (GMR = 0.33; 95% CI: -0.15, 0.76; p-value = 0.009) attributable to HIV positivity with a non-significant reduction in Inaba GMR by about 50% due to HIV positivity. When doubling of retinol levels modelled, GMR reduction against Ogawa were non-significant but that against Inaba resulted in a significant reduction in geometric mean titer (GMT) (GMT-0.33, C.I 0.16–0.66, p-value 0.002). At 1000copies/ml viral load cut off and 350 cells/μl CD4 counts, Ogawa GMT was two times higher 11.16 (95%CI: 8.20–15.19) versus 6.06 (95%CI: 4.04–9.10) in low viremia participants, and three times higher in above threshold CD4 count participants; 24.81 (95%CI: 18.94–32.50) versus 7.07 (95%CI: 5.22–9.58).

**Conclusion:**

Our results show that while Shanchol™ is immunogenic in both HIV+/- individuals, HIV + participants responded poorly. Viral load and CD4 count affected vaccine immunogenicity. More research is required for detailed understanding of this in order to appropriately inform policy and practice.

## Introduction

Cholera is an acute watery diarrheal disease caused by *Vibrio cholerae* serogroups O1 and O139. Serogroup O1 is divided into two major serotypes of Inaba and Ogawa [1]. Cholera outbreaks are common in endemic settings, which also tend to have the world’s poorest and vulnerable populations [2,3]. The disease affects at least 47 countries worldwide with an estimated 2.9 million cases and about 95,000 annual deaths [4]. However, these figures only account for about 5–10% of the actual number as most of the cases go unreported [5].

Sub-Saharan Africa remains a hub of cholera outbreaks since 1970 with Zambia reporting its first outbreak in 1977 [6]. Since then, Zambia has had recurrent outbreaks occurring almost yearly [7]. According to the WHO country stratification on the incidence rate and fatality rate, Zambia is classified in the (E) region, which is a region with high child and very high adult mortality attributed to cholera [5]. The Lukanga Swamps (study site), is located in Central Province of Zambia and often records recurrent cholera outbreaks. For instance, in 2013 and 2014, the area recorded 47 and 41 cholera cases respectively while in 2016 alone, a total of 27 cases with two fatalities were recorded [8].

A review of the burden of HIV in lower-to-middle income countries (LMIC) reported an estimated 33.4 million HIV infected individuals living in LMICs [9]. As of 2018, approximately 1.2 million people were living with HIV in Zambia according to a report by UNAIDS [10]. A study carried out in Mozambique reported that people living with HIV have a high prevalence of Cholera (23%) compared to the control group (13%) [11].

Vaccination with oral cholera vaccines (OCV) is an effective way to reduce the high cholera incidence when deployed in combination with improved water, sanitation and hygiene (WASH) facilities [6].

Poor nutrition resulting in malnutrition has been known to affect the immune responses to OCV in individuals living in LMIC [12]. Nutritional status such as retinol levels, influence innate immune cells and epithelial cells associated with mucosal surfaces, thus in the presence of retinol, epithelial cells express retinaldehyde dehydrogenase which enhances antibody production by stimulated B-cells [13–15]. However, in settings of poor nutrition coupled with a high prevalence of HIV infection, it is not clear how these interact to affect OCV uptake. In this study, we evaluated the independent effects of HIV infection and serum retinol level as a derivative of Vitamin A on immunogenicity to Shanchol™ in a cholera endemic setting.

## Materials and methods

### Study setting

The Lukanga Swamps (260,000 hectares, 14°24’S 027°38’E), located in the Central Province of Zambia, is one of the cholera hotspots with recurrent annual cholera outbreaks before the Shanchol™ intervention in 2016. This area has shallow swamps and several lagoons and makes up the largest permanent water body in the Kafue basin. The area has approximately 16,000 inhabitants and the major economic activity of the population is fishing.

### Study design and participants

This was an observational study nested on a cohort study aimed at profiling immunological characteristics of a population at risk of cholera before and after receiving 1^st^ and 2^nd^ dose of OCV (Shanchol™) intervention. The parent cohort study was part of the Zambian Ministry of Health’s cholera prevention and control program, which deployed Shanchol™ and WASH interventions to the affected population in Lukanga Swamps in 2016. A total of 223 adults aged 18 to 65 years, living in the study area, and available for the duration of the study were enrolled in the study and were followed up for 4 years. Adults who had medical conditions such as hepatic disease, diarrhoea within the previous 7 days or a history of persistent diarrhoea (defined as diarrhoea that lasts for 14 days or longer), and ever having received an OCV were excluded. This parent study has been registered as a clinical trial on clinical trials.gov with trial # NCT04423159. Of the 223, only 47 consented to voluntary HIV testing and these are the ones included in our study.

Ethical approval was obtained from the University of Zambia Biomedical and Research Ethics Committee (UNZABREC) reference number 007-12-16. Written informed consent was obtained from all participants of the study.

### Procedures

#### Vaccine administration and blood sample collection and processing

The vaccine was stored at a temperature of 2–8°C prior to administration. The first dose of 1.5 ml OCV was given at baseline (day 0) and the second dose on day 28. Both doses were administered orally. Up to 10mls of venous blood was obtained from each participant, in vacutainers, before vaccination (day 0) and on day 28 followed by 6, 12, 24 and 30 months after the first dose, serum was separated and stored at -20°C until laboratory testing.

#### Retinol quantification

Retinol quantification was performed by using High-Performance Liquid Chromatography (HPLC) [16]. The quantification was done on the SHIMADZU Prominence HCT2010 HPLC system. Briefly, 100μl of serum sample/control was mixed with an equal volume of an ethanolic retinyl acetate mixture in a clean capped glass tube to release the retinol from its complex. The retinyl acetate served as the internal standard for the analysis. The resulting mixture was vortexed for 30 seconds. The released retinol was then recovered from the ethanol solution by two extractions of 500μl each of n-hexane mixed into a separate capped tube. The combined hexane extracts were evaporated to dryness under a gentle stream of nitrogen gas. Thereafter, the resulting residue was then dissolved in 100μl of ethanol. A volume of 60 μl of this residue solution was applied on the reverse phase HPLC column (5-μm C18, 25-cm) protected by a Supelcosil^TM^ C-18 guard column and eluted at 1.5 ml/min, using a 98:2, methanol: water solvent, with detection at 325nm.

#### HIV testing

The blood-based HIV test kit, Alere Determine^™^ HIV1/2 (Alere Scarborough, Inc.) was used to screen for HIV. A second confirmatory test, the Uni-Gold™ HIV (Trinity Biotech Manufacturing Ltd) was used and if the results from both tests were discordant a tie-breaker test SD Bioline (Standard Diagnostics, Inc) was used.

#### CD4+ T lymphocytes enumeration

To enumerate percentage and absolute cell counts of CD4+ T lymphocytes, the FACS Calibur instrument was used using lysed whole blood collected in vacutainers with EDTA. 50μl of the sample was stained directly in a BD Trucount tube. Fluorescent beads were released by the lyophilized pellet in the tube as it dissolved. During analysis, the absolute number (cells/μl) of positive cells in the sample was determined by comparing cellular events to bead events. The BD Multset software was used to determine the number of positive cells per microliter of blood (absolute counts).

#### HIV-1 viral load testing

Human plasma collected in EDTA anticoagulant was used for viral load analysis. HIV-1 viral load testing was performed using the COBAS^®^Ampliprep/ COBAS^®^Taqman^®^ 48 HIV-1 Test. The specimen was prepared using the automated COBAS® AmpliPrep Instrument with amplification and detection was done using the COBAS® TaqMan® 48 Analyzer as per manufacturer’s instructions.

#### Cholera vibriocidal assay

The vibriocidal responses were assessed according to previously described methods [17] with some modifications. The strains were incubated with heat-inactivated serum and exogenous guinea pig complement (Sigma Aldrich S1639-5ML) at 37°C for 1 hour, shaking (50 revs/min). Vibriocidal titers were defined as the reciprocal of the highest serum dilution resulting in a 50% reduction in optical density (595 nm) compared to controls without serum. Standard monoclonal antibody (mAb) (Boston, MA, USA, CF29.1.A2) and high titer pooled sera were used to normalize the results in case of inter-assay variations. Seroconversion was defined as a 4-fold or greater increase in vibriocidal titers after vaccination in comparison to the baseline (D0) titers with a titer of 5 assigned in cases where no vibriocidal activity was observed.

### Sample size calculation

**Fig 1:**
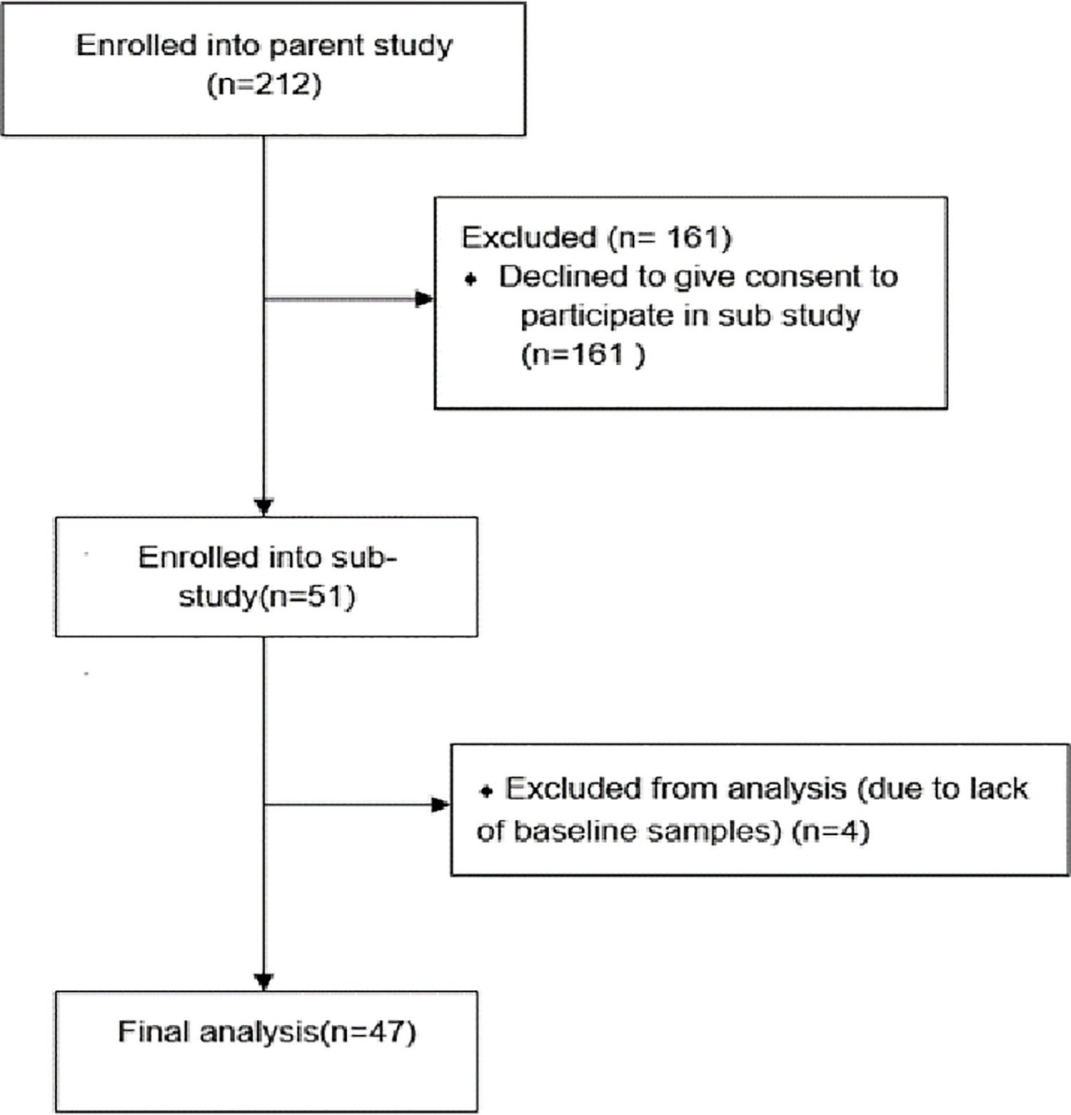
Flow diagram of the participants who took part in the study.

The participants of this study were a subset of a larger cohort study that has been described above. The sample size for the large study was calculated to be 176 with 95% confidence (α = 0.05 (two tailed)), 80% power (β = 0.2), a difference of 0.3 and conservative estimates of 0.5 variance for pre-vaccine and post-vaccine groups. Adding 20% to account for anticipated attrition yields a total required sample size of 212. Thus, for the parent study, 212 participants were enrolled. This current study excluded individuals from the parent study who did not consent to HIV testing (161) or had no baseline titers (4) (Fig 1). Therefore, a total of 47 participants were included in this study.

### Statistical analysis

The primary outcome was geometric mean titer of Vibriocidal immune responses. The titers were transformed using natural logarithm prior to statistical analyses. Participants’ socio-demographic and clinical characteristics were presented as frequencies (percentages) and mean (standard deviation). Geometric mean titres and 95% confidence intervals were calculated for key background characteristics. The repeated measurements of the outcomes introduce panel structure into the dataset. Therefore, random-effects log-normal regression model (i.e. ‘**xtgee**’ gaussian-family; identity-link command in Stata) was used to estimate the independent effects of HIV status and Retinol on immune response to OCV, adjusting for age, sex, education and occupation. The participant ID represents the panel and the measurement occasions represent the time variable. For the secondary outcome of seroconversion, random-effects log-binomial model (i.e. ‘**xtlogit**’ with ‘**margins**’ in Stata) was used to estimate the effects of HIV status and Retinol on seroconversion controlling for the confounding effects of age, sex, education and occupation. In all the models, Retinol was transformed into log base 2 so that the effect is interpreted as doubling in retinol levels. Level of statistical significance was set at a 2-tailed p-value of 0.05 or less. Data analysis was performed using Stata 14.2 for Windows (StataCorp, College Station, TX, USA).

## Results

### Characteristics of participants

A total of 47 adults were included in the analysis. Of these, 42 (89%) were females. The median age was 38 years (IQR = 31–45). More than half of the participants had an education level of grade 8–12 (56%). The major occupation engaged in by the participants was fishing (77%). Among the HIV+ participants, 15(65%) had CD+4 count of 350cells/μl or more while 78% had a viral load of 1000 copies/ml or less (Table 1).

**Table 1:** Geometric mean ratios of *V*. *cholerae* O1 Ogawa and *V*. *cholerae* O1 Inaba by background characteristics of participants.

Characteristics	Number (% of total)	*V cholerae* O1 Ogawa	*V cholerae* O1 Inaba
		GMTs (95% CI)	GMTs (95% CI)
**Sex**			
** Female**	5 (11)	25.67 (12.16–54.20)	8.24 (5.43–12.50)
** Male**	42(89)	16.10 (13.17–19.66)	11.05 (9.18–13.30)
**Age(years)**			
** ≤35**	16 (34)	19.81 (13.57–28.92)	12.60 (8.61–18.44)
** >35**	31 (66)	15.76 (12.55–19.78)	9.91 (8.29–11.85)
**Education^¶^**			
** Grade 1–7**	16(44)	19.82 (14.03–28.01)	11.53 (8.61–15.44)
** Grade 8–12**	20(56)	13.75 (10.43–18.13)	11.68 (8.72–15.66)
**Occupation^¶^**			
** Fishing**	34(77)	17.38 (13.77–21.95)	12.69 (10.18–15.81)
Others*	10(23)	14.74 (9.58–22.68)	7.07 (5.56–8.99)
**Viral loads (copies/ml)^¶¶^**			
** >1000**	4 (22)	6.06 (4.04–9.10)	6.80 (4.95–9.35)
** ≤1000**	14 (78)	11.16 (8.20–15.19)	6.76 (5.63–8.10)
**CD4count (cells/μl)^¶¶^**			
** <350**	8(35)	7.07 (5.22–9.58)	7.33 (5.42–9.93)
** ≥350**	15(65)	24.81 (18.94–32.50)	7.18 (5.84–8.83)
**Total**	**47 (100)**	**16.95 (13.94–20.60)**	**10.70 (9.02–12.69)**

^¶^Total was not equal to 47 due to missing information.

^¶¶^Total was not equal to 24 due to missing information.

Others* refers to traders, farmers and students.

GMT refers to Geometric Mean Titre, CI (confidence interval).

Overall, the geometric mean titres (GMTs) of *V cholerae* O1 Ogawa and Inaba were [16.95 (95%CI: 13.94–20.60)] and 10.70 [(95%CI: 9.02–12.69)] respectively (Table 1). Ogawa GMT was higher among female participants [25.70 (95%CI: 12.16–54.20)] than male participants [16.10 (95%CI: 13.17–19.66)] and those 35 years old or younger [19.81 (95%CI: 13.57–28.92)] than those older than 35 years old [15.76 (95%CI: 12.55–19.78)]. Study participants who completed grades 1–7 [19.82 (95%CI: 14.03–28.01)] had higher Ogawa GMT than those who had grades 8–12 [13.75 (95%CI: 10.43–18.13)] while those who engaged in fishing [17.38 (95%CI: 13.77–21.95)] also had higher Ogawa GMTs compared to those involved in other occupation [14.74 (95%CI: 9.58–22.68)].

Ogawa GMT was about two times higher in participants with viral loads of 1000copies/ml or less [11.16 (95%CI: 8.20–15.19)] compared to those with more than 1000 copies/ml [6.06 (95%CI: 4.04–9.10)] and about three times higher in participants with CD4 counts of 350 cells/μl or more [24.81 (95%CI: 18.94–32.50)] compared to those with CD4 counts lower than 350 cells/μl [7.07 (95%CI: 5.22–9.58)] (Table 1). Inaba GMT was higher in male participants [11.05 (95%CI: 9.18–13.30)] than the females [8.24 (95%CI: 5.43–12.50)] but lower in the older ones [9.91 (95%CI: 8.29–11.85)] than the young [12.60 (95%CI: 8.61–18.54)]. Inaba GMT does not vary widely by education, viral load and CD4 count, however, participants whose primary occupation was fishing [12.69 (95%CI: 10.18–15.81)] had higher Ogawa GMTs (about two times more) compared to those involved in other occupation [7.07 (95%CI: 5.56–8.99)]. Despite the observed trends the GMT results were not statistically significant.

### Effects of HIV status and retinol on the immune response to OCV

After adjusting for the confounding effects of age, sex, occupation, and education, there was a statistically significant reduction in Ogawa GMR by 67% due to HIV positivity (GMR = 0.33; 95% CI: -0.15, 0.76; p-value = 0.009) (Table 2). Similarly, there was a reduction in Inaba GMR by about 50% due to HIV positivity, but this is likely to be due to chance as it was not statistically significant (GMR = 0.46; 95%CI: 0.19, 1.15; p-value = 0.096) (Table 2). In the HIV positive group, CD4 cell counts and viral loads did not have any significant effects on responses to OCV (S1 Table). For retinol level, there was a reduction in Ogawa GMR due to a doubling of retinol level but again this was not statistically significant (GMR = 0.86; 95%CI: 0.40, 1.86; p-value = 0.698) while for Inaba the doubling of retinol level resulted into a significant reduction in GMR (GMR = 0.33; 95%CI: 0.16, 0.66; p-value = 0.002) (Table 2).

**Table 2:** Effects of HIV status and retinol on the immune response to OCV.

**HIV Status**	** **	***V cholerae* O1 Ogawa**			
	**Number (% of total)**	**Crude GMR (95%CI)**	**P-value**	**Adjusted GMR (95% CI)** *	**P-value**
** Negative**	23(49)	Ref		Ref	
** Positive**	24(51)	0.46 (0.23–0.93)	0.029	0.33 (0.15–0.76)	**0.009**
**Retinol (μmol/l)** **					
** Mean ± SD**	3.14 ± 0.64	1.10 (0.63–1.94)	0.73	0.86 (0.40–1.86)	0.698
		***V cholerae* O1 Inaba**			
**HIV Status**	**Number (% of total)**	**Crude GMR (95%CI)**	**P-value**	**Adjusted GMR (95% CI)** *	**P-value**
** Negative**	23(49)	Ref		Ref	
** Positive**	24(51)	0.49 (0.27–0.92)	0.026	0.46 (0.19–1.15)	0.096
Retinol (μmol/l)**		Ref		Ref	
** Mean ± SD**	3.14 ± 0.64	0.65 (0.39–1.07)	0.07	0.33 (0.16–0.66)	**0.002**

*Adjusted for sex age, education, & occupation

**Transformed to log2, GMR: Geometric Mean Ratio, CI: Confidence Interval.

### Kinetics of vibriocidal geometric mean titers against Ogawa and Inaba by HIV status

**Fig 2:**
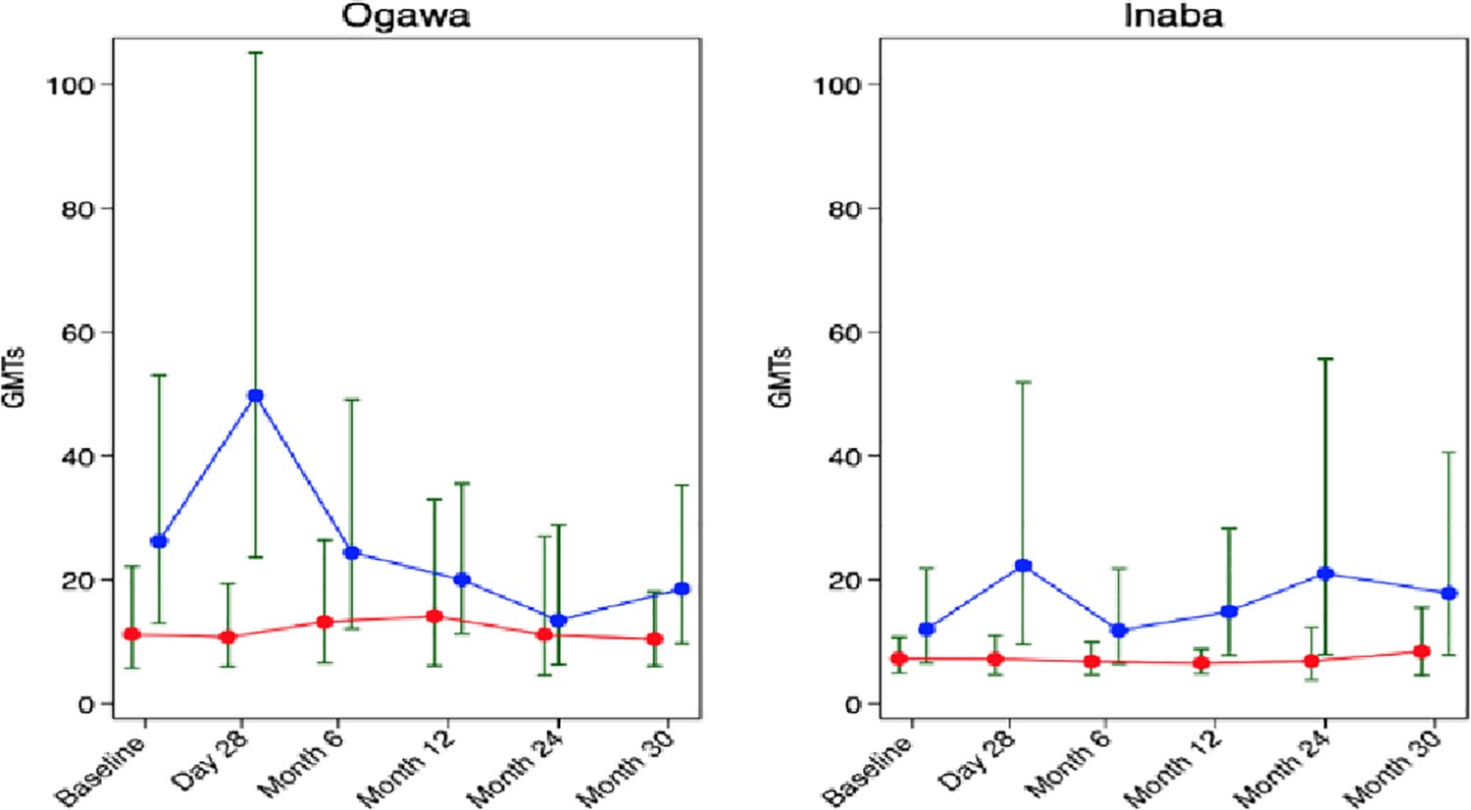
Kinetics of vibriocidal geometric mean titers (95% CI) against Ogawa and Inaba by HIV status. Red dots indicate HIV + participants and blue dots HIV- participants.

The GMTs against Ogawa and Inaba are shown in Fig 2 below. Against both serotypes, HIV–participants had higher GMTs than HIV + participants across all the time points (DO, D28, M6, M12, M24, M30). The only significant differences in GMTs were at D28 for Ogawa (HIV- 49.79, HIV + 10.76, p-value 0.002) and for Inaba D28, M2 and M24 were significant with GMTs of (22.31, 7.20 p = 0.02), (14.86, 6.55 p = 0.03) and (21.01, 6.89 p = 0.04) respectively.

### Effects of HIV status and retinol on seroconversion

HIV + individuals compared to the negatives have about 10% lower risk of seroconverting to O1 Ogawa after vaccination although this association is likely to have occurred by chance (RR = 0.90; 95%CI: 0.22, 3.63; p-value = 0.877). Similarly, against Inaba, HIV + respondents are about 2 times less likely to seroconvert after vaccination compared to those who are negative but this association was not statistically significant (RR = 0.50; 95%CI: 0.11, 2.40; p-value = 0.375). A doubled level of retinol reduced the risk of sero-conversion to O1 Ogawa and Inaba by 8% and 12% respectively, although both associations were not statistically significant (RR = 0.92; 95%CI: 0.77, 1.10; p-value = 0.375 & RR = 0.88; 95%CI: 0.72, 1.08; p-value = 0.227) (Table 3).

**Table 3:** Percent seroconversion by HIV status and retinol level.

**HIV Status**	** **	***V cholerae* O1 Ogawa**			
	**Number (% of total)**	**Crude RR (95%CI)**	**P-value**	Adjusted RR (95% CI)*	**P-value**
** Negative**	23(49)	Ref		Ref	
** Positive**	24(51)	0.88 (0.32–2.39)	0.801	0.90 (0.22–3.63)	0.877
Retinol (μmol/l)**					
** Mean ± SD**	3.14 ± 0.64	0.96 (0.85–1.09)	0.553	0.92 (0.77–1.10)	0.368
		***V cholerae* O1 Inaba**			
**HIV Status**	**Number (% of total)**	**Crude RR (95%CI)**	**P-value**	Adjusted RR (95% CI)*	**P-value**
** Negative**	23(49)	Ref		Ref	
** Positive**	24(51)	0.37 (0.10–1.36)	0.127	0.50 (0.11–2.40)	0.375
Retinol (μmol/l)**					
** Mean ± SD**	3.14 ± 0.64	0.96 (0.85–1.09)	0.551	0.88 (0.72–1.08)	0.227

RR = Crude Risk Ratio, RR = Risk Ratio, CI = Confidence Interval, Ref = reference *Adjusted for sex age, education, & occupation.

## Discussions

A majority of the participants enrolled in this study were fishermen; these were at higher risk of cholera infection due to their way of life such as practicing open defecation in the water lagoons that are later used for fetching drinking water. A study in Uganda reported Cholera as the leading cause of morbidity and mortality in Ugandan fishermen [18]. There was a difference in sex distribution among the participants with 10.6% being female and 89.4% male participants as shown in Table 1. This is not a surprising finding for the Zambian setting because fishing is mostly dominated by men in most fishing camps and areas. Despite the unequal distribution of sex, it had no cofounding effects on the reported outcomes of immunogenicity and seroconversion.

A noticeable trend, although not statistically significant, pointed towards HIV negative participants having higher vibriocidal GMTs from baseline up until month 30 compared to HIV positive individuals, against both serogroups (Fig 2). This trend is in agreement with what was observed in Haitian adults [19]. It’s also consistent with the reports on immune response in the HIV/AIDS population to other vaccines as this population generally has lower antibody titers as compared to healthy individuals [20]. This could be explained by susceptibility to severe disease occurring in HIV infected individuals when secretory IgA, which is supposed to agglutinate the vibrios by binding to the surface antigens, therefore, preventing them from attaching to the mucosal membranes, is depleted [11].

Also, our study observed a better response in our target population against the Ogawa than Inaba serotype. One possible explanation for this is the relative fluctuation of the two serotypes (Ogawa and Inaba) in an endemic population between epidemics of the disease at a given time-point were one of the two serotypes being responsible for the majority of cases [21,22]. The *V*. *cholerae* serotypes have been reported to undergo serotype conversions in both directions in most of these endemic areas [23]. Zambia had a cholera outbreak in 2017 and this was predominantly caused by the 01 Ogawa serotype [6]. Given that CD4+ T cell priming is a critical step in vaccination as there is a direct relationship between CD4+ cells and protective immunity and long term humoral responses [24], we hypothesized that the population could have been better primed against Ogawa as compared to Inaba resulting in the observable differences in the trends of GMTs between the two serotypes.

Micronutrients such as vitamin A have been reported to play a crucial role in immune regulation [25,26]. Retinol a derivative of vitamin A has been shown to increase antibody response to T-cell-dependent and T-cell-independent antigens and restoration and maintenance of mucosal function as well as integrity [27]. While retinol supplementation has been known to reduce the severity and pathogen-induced mortality, also at the same time, retinol deficiency leads to impaired mucosal and systemic immune responses henceforth affecting uptake of orally administered vaccines [28,29]. We analyzed retinol levels before administration of Shanchol™ and the results indicated that all our participants had serum retinol levels above 0.7umol/L, a cut-off for retinol deficiency [30].

When the retinol levels were doubled, our study reveals a reduction in GMRs across both serotypes which is contrary to what has been reported on the effects of retinol supplementation on efficacies of other vaccines. For instance, a study elsewhere reported the use of vitamin A as an adjuvant in oral vaccines increasing the vaccine efficacies in settings that have reported low efficacies such as Asia, Latin America and Africa [31]. Our findings might suggest that retinol supplementation during the administration of Shanchol™ in the HIV infected population might not be of significant benefit.

Our study has shown a lack of statistical significance in differences in the seroconversion rates among the HIV- and the HIV + participants following uptake of the OCV Shanchol™. In addition, our analysis of results showed that there was no appreciable boosting in vibriocidal titers following the second dose at 28 days after the first dose in this cohort (Fig 2). This is compatible with what previous studies reported over the lack of benefit of a second dose [32,33]. A hypothesis that could explain this lack of significant impact of the second dose, is that the first dose elicits an immune response in the intestinal mucosal, thus this response possibly blocks the uptake of the second dose [33].

## Limitations

Our study had some limitations. Firstly, the assay is limited in its inability to detect re-exposure; therefore, re-exposure to cholera could have been missed if it did occur and shortly waned off. Secondly, we had several participants that did not show up at all the time-points. We underestimated how mobile fishermen can be, hence the missing data was excluded in some of the analysis, and therefore, going forward this should be put into consideration in such populations.

Thirdly, the second dose was given 28 days past the first dose, which was a deviation from the recommended dose intervals as per manufacturer’s instructions. This deviation was due to logistical challenges in getting back to the vaccinated population which is in a remote setting after the recommended 14 days coupled with limited resources. It was going to be interesting if we included a randomized controlled trial sub-study of 14 days versus 28 days Shanchol™ administration so that we could have answered which interval is superior.

Lastly, this study did not look at how other immunological factors like cytokines of the HIV -/+ participants and how these could have affected immunogenicity to the vaccine as these have been reported to qualitatively contribute to different immune responses. Hence, going forward for similar studies, we recommend that such factors be put into consideration when assessing vaccine immune responses. Thus, despite us observing non-significant results in our study, we observed a trend which can guide a hypothesis to be explored with a larger sample size and also by looking at other immunological parameters as mentioned above.

## Conclusion

Our study has demonstrated that the OCV Shanchol™ can elicit an immune response in the HIV+ populations. The observed immunogenicity was dependent on the serotype, the amounts of CD4 + T cells as well as viral load, hence these parameters should be put into consideration when developing as well as deploying a vaccine for this vulnerable population. In addition, the role played by retinol in Shanchol™ immunogenicity was unclear. Also, our study suggests that supplementing with retinol may not be beneficial in OCV uptake as we observed a reduction in GMRs when the retinol amounts were doubled.

## Supporting information

S1 Table Effects of viral load and cd4 count on immune response to OCV. (DOCX)
