# Supplementary material for: Effect of HIV status and retinol on immunogenicity to oral cholera vaccine in adult population living in an endemic area of Lukanga Swamps, Zambia
Source: PLoS One. 2021 Dec 2;16(12):e0260552. doi: 10.1371/journal.pone.0260552 (PMC8639067; doi:10.1371/journal.pone.0260552)
Supplement: S1 Table — (DOCX) [file pone.0260552.s001.docx]

| S1 Table. Effects of viral load and cd4 count on immune response to OCV | | | | | |
| --- | --- | --- | --- | --- | --- |
| Viral load(copies/ml) |  | ***V cholerae O1 Ogawa*** | | | |
|  | **Number (% of total)** | **Crude GMR (95%CI)** | **P-value** | **Adjusted GMR (95% CI)*** | **P-value** |
| >1000 | 4 (22) | Ref |  | Ref |  |
| ≤1000 | 14 (78) | 1.79 (0.57 - 5.62) | 0.316 | 1.45 (0.97 - 2.16) | 0.07 |
| CD4 count (cells/µl) | |  |  |  |  |
| <350 | 8(35) | Ref |  | Ref |  |
| ≥350 | 15(65) | 2.08 (0.84 - 5.17) | 0.115 | 1.24 (0.61 - 2.53) | 0.554 |
|  |  | ***V cholerae* O1 Inaba** | | | |
| Viral load(copies/ml) | **Number (% of total)** | **Crude GMR (95%CI)** | **P-value** | **Adjusted GMR (95% CI)*** | **P-value** |
| Negative | 4 (22) | Ref |  | Ref |  |
| Positive | 14 (78) | 0.99 (0.66 - 1.50) | 0.968 | 1.04 (0.75 - 1.45) | 0.82 |
| CD4 count (cells/µl) | |  |  |  |  |
| <350 | 8(35) | Ref |  | Ref |  |
| ≥350 | 15(65) | 0.98 (0.67 - 1.43) | 0.906 | 0.89 (0.64 - 1.23) | 0.471 |

*Adjusted for sex,age, education, & occupation, GMR: Geometric Mean Ratio, CI: Confidence Interval
